# Association between Life Skills and Academic Performance in Adolescents in the Autonomous Community of Aragon (Spain)

**DOI:** 10.3390/ijerph18084288

**Published:** 2021-04-18

**Authors:** Beatriz Sánchez-Hernando, Raúl Juárez-Vela, Isabel Antón-Solanas, Ángel Gasch-Gallén, Pedro Melo, Tam H. Nguyen, José Ramón Martínez-Riera, Elisa Ferrer-Gracia, Vicente Gea-Caballero

**Affiliations:** 1 Centro de Salud Amparo Poch, Servicio Aragonés de Salud, C/Emilia Pardo Bazán, s/n, 50018 Zaragoza, Spain; beasanhern@gmail.com; 2 Grupo Enfermero de Investigación en Atención Primaria de Aragón (GIIS094-GENIAPA), Instituto de Investigación Sanitaria de Aragón, Avda. San Juan Bosco 13, 50009 Zaragoza, Spain; angelgasch@unizar.es; 3 Department of Physiatrics and Nursing, University of Zaragoza, C/Domingo Miral s/n, 50009 Zaragoza, Spain; 4 Department of Nursing, University of La Rioja, Centro Investigación Biomédica de la Rioja CIBIR-GISOSS, C/ Duquesa Victoria 88, 26004 Logroño, Spain; raul.juarez@unirioja.es; 5 Institute of Health Sciences/School of Nursing (Porto)/Centre for Interdisciplinary Research in Health, Universidade Catolica Portuguesa, 4169-005 Porto, Portugal; pmelo@porto.ucp.pt; 6 NursID Project, Center for Health Technology and Services Research, 4200-450 Porto, Portugal; 7 William F. Connell School of Nursing, Boston College, Newton, MA 02467, USA; tam.nguyen@bc.edu; 8 Departamento Enfermería Comunitaria, Medicina Preventiva y Salud Pública e Historia de la Ciencia, University of Alicante, E-03080 Alicante, Spain; jr.martinez@ua.es; 9 Sección de Promoción de la Salud, Departamento de Sanidad, Dirección General de Salud Pública, Vía Universitas 36, 50017 Zaragoza, Spain; eferrerg@aragon.es; 10 Nursing School La Fe, adscript center of Universidad de Valencia, Research Group GREIACC, Health Research Institute La Fe, Avda. Fernando Abril Martorell 106, 46026 Valencia, Spain; gea_vic@gva.es

**Keywords:** social skills, self-efficacy, emotions, academic performance, adolescents, health promotion

## Abstract

Background: Learning and socio-emotional development is promoted through the creation and nurturing of an optimal school climate. This study aims to analyze the relationship between life skills and academic performance in a large sample of adolescents from the autonomous community of Aragón (Spain). Methods: A cross-sectional study was conducted on the life skills and academic performance of a sample of 7th and 8th grade middle school students during the academic year 2018–2019. A sample of 43 middle schools were randomly selected; the final sample comprised 1745 students. The following data were collected through an anonymized, previously validated questionnaire: sociodemographic variables, social skills, self-efficacy, affective balance, and academic performance. Results: We found a statistically significant association between life skills and academic performance (*p* < 0.001) in our sample. We also observed significant gender differences in life skills, with boys obtaining higher scores in cognitive skills and affective balance, and with girls achieving higher scores in social skills. Conclusion: We argue that life skills should be integrated into educational policies in order to improve the academic performance and health outcomes of students.

## 1. Introduction

Academic performance is an indicator of the success of the teaching and learning process. According to Solano [1], academic performance is concerned with the assessment of students’ knowledge on a particular subject. It is a complex construct that is influenced by numerous components, including personal (cognitive capacity and personality) and educational factors [2]. In addition, academic performance is associated with social and psychological wellbeing. One way to address these factors is through life skills [3].

Life skills are a set of psychosocial abilities that allow the individual to act competently and behave adequately in a range of day-to-day situations and scenarios [3]. According to Morales-Rodríguez et al. [3], there are three types of life skills: social, cognitive, and affective. Social skills are defined as the specific social abilities that are necessary to interact with, and relate to, others in an efficient, respectful, and mutually satisfactory manner [4]. Cognitive skills are the foundation for the construction and organization of knowledge and reasoning [5] and include abilities such as self-efficacy, defined as the belief in one’s ability to face diverse situations [6,7,8]. Affective skills influence subjective wellness; subjective wellness could be colloquially described as happiness and comprises two components, namely cognitive and emotional. In order to achieve a high level of subjective wellness, individuals must have a high level of personal satisfaction (cognitive component) and positive affective balance (emotional component) [9].

An educational approach which is based on life skills provides a strong theoretical base for the design and implementation of intervention programs to improve adolescents’ ability to face life challenges [3,10]. In fact, when emotional education, including thinking, emotions, and behavior, is integrated into these programs, an improvement in the participants’ social and affective competencies, as well as their academic performance, is observed [11,12]. Specifically, promoting adolescents’ sense of coherence, and thus also self-efficacy and coping mechanisms, can improve their capacity to face adversities and achieve a higher level of subjective wellness [13,14].

The educational context offers students the opportunity to learning not only theoretical knowledge but also attitudes, habits, and social skills. Creating and maintaining a positive learning environment supports student learning and offers opportunities for social and emotional development [11]. For example, the kind of multifactorial interventions implemented at health promoting schools can create favorable conditions for learning and contribute to the students’ psycho-affective development, defined as the process through which children construct their identity, self-esteem and self-confidence [15,16,17]. Health promoting schools are based on a model of participative education and integrate health promoting interventions and activities [15] into their curricula.

During adolescence, young people undergo numerous emotional changes that contribute to developing and shaping their personality; in this crucial period of their lives, adolescents may need support in order to act competently and responsibly. Acting in a competent and responsible way is important, as it may affect their personal life as well as their academic performance. There is a positive association between academic performance and life skills in the general population. However, the results from previous investigations in this field are difficult to interpret. Some authors [18,19,20] have suggested that a positive association exists between academic performance and social skills such as self-esteem and empathy. However, the level of empathy and assertiveness ranges from good in some studies [18], to moderate-high [3,21] and moderate-low [22] in others. Similarly, a positive association exists between academic performance and self-efficacy [8,23,24,25,26]. The relationship between academic performance and affective balance, meanwhile, is unclear in the literature. Some studies [11,24,27,28,29] suggest that a positive association exists between them, whereas others [30] deny it. Furthermore, according to Hayat et al. [24], academic performance is associated with self-efficacy and positive emotions. 

Thus, this study aims to identify the life skills of a sample of 7th and 8th grade students in a total of 43 middle schools in the region of Aragón (Spain), and study the association between said skills and the students’ academic performance. As a secondary aim, we will describe gender differences in the students’ life skills. 

## 2. Materials and Methods

### 2.1. Design

A cross-sectional study was carried out during the 2018–2019 academic year.

### 2.2. Population and Sample

Our target population comprised all the 7th and 8th grade (equivalent to 1st and 2nd ESO in the Spanish educational context) students registered at one of the 185 mainstream middle schools in the Spanish region of Aragón (*n* = 27,184) [31]. We excluded schools dedicated to providing an education for students with a special educational need or disability and grouped rural educational centers. A minimum sample of 379 students was estimated for sample size calculation, with a confidence level of 95% and an error margin of 5%. We used a conglomerate sampling technique whereby we randomly selected 43 mainstream middle schools from among a total of 185 educational centers using the tool “Research Randomizer” (www.randomizer.org; accessed on April 2017); a total of 5132 7th and 8th grade students were registered in the selected schools. Subsequently, we approached all 5132 7th and 8th grade students and requested their consent to participate in the study, as well as that of their parents or legal tutors. We excluded students who could not speak Spanish from the final sample. A total of 1745 students gave their informed consent to participate in the study and completed the questionnaire (a rate of response of 34%).

### 2.3. Data Collection

The participants completed a self-administered, anonymous questionnaire during April 2019. The following variables were quantified: sociodemographic characteristics (sex, grade, age, number of siblings, sibling position, cohabitants, father’s level of education, mother’s level of education, weight, height and perceived level of health), cognitive, social, and affective life skills, and academic performance. Specifically, life skills were measured using previously validated tools including the questionnaire from the Health Behavior in School-aged Children (HBSC) study [32], the general self-efficacy scale [33], the first subscales of the social skills assessment scare [34], and the affective balance scale [9].

The final self-administered questionnaire was adapted and validated using the expert panel technique [35]. Inclusion criteria to join the panel were: (a) being a qualified nurse or doctor, (b) having at least 5 years post-registration experience in public health and/or community health, (c) having taken part in at least 5 health promotion interventions in educational centers. A total of 6 experts took part in two group sessions lasting approximately 2 h each in December 2017. Internal consistency of the adapted version of the questionnaire was examined using a Cronbach’s alpha coefficient for each of the dimensions resulting from confirmatory factor analysis (CFA). Internal consistency or homogeneity of the new version of the tool was good with a Cronbach’s alpha coefficient of 0.8465; the separate values obtained for each of the factors was close to one, which indicated that factor analysis for each of the factors was consistent. Factor analysis with 6 factors explained 75.25% of the model variance. The instrument’s validity was evaluated using different indicators (NFI = 0.802; RMSEA = 0.067; CFI = 0.891; SRMR = 0.093) and showed good adjustment.

The validated version of the instrument comprised 53 items classified into 5 subscales, namely sociodemographic variables (10 items), social skills (14 items), self-efficacy (10 items), affective balance (18 items), and academic performance (1 item). The subscale sociodemographic variables included aspects measured in previous, similar studies [32]; the subscale social skills was adapted from the social skills assessment scale [34]; the subscale self-efficacy consisted of the general self-efficacy scale [33]; the subscale affective balance consisted of the affective balance scale [9]; and finally, the subscale academic performance included the variable final grade, obtained by calculating the mean score of each of the first and second trimester subjects. 

The subscale ‘social skills’ was measured using a 5-point Likert scale analyzing the frequency of certain behaviors ranging from never (1) to always (5); the subscale ‘self-efficacy’ was measured using a 4-point Likert scale identifying the degree of applicability of certain statements ranging from untrue of me (1) to true of me (4); the subscale ‘affective balance’ was measured on a 3-point Likert scale ranging from never or almost never (1) to always or almost always (3), quantifying the frequency with which the adolescents experienced certain emotions. The students’ academic performance was assessed using the students’ average score for the whole academic year; in Spain, the academic score is a number from 0 to 10.

### 2.4. Data Analysis 

Data codification, processing, and analysis were completed using the statistical software STATA/SE v16.0. (StataCorp. 2020, College Station, TX, USA). Categorical variables were presented using frequencies and percentages; numerical variables were presented using mean and standard deviation. All the variables followed a normal distribution with the exception of self-efficacy and affective balance. We used the chi-square test to examine the relationship between categorical variables (independent variables with the dependent variable academic performance). The main estimates were presented with a 95% confidence interval, a margin of error of 5%, and a level of statistical significance *p* < 0.05. 

### 2.5. Ethical Considerations

The data were dissociated to ensure that the information was treated confidentially and anonymously, following the Data Protection Regulation (EU) 2016/679 of the European Parliament and the Spanish Organic Law 3/2018. The researchers did not declare any type of ethical, moral, or legal conflict, nor did they claim to have received financial compensation of any kind. The participants did not receive any type of compensation for answering the questionnaire, as it was voluntary. The project was endorsed by the General Directorate of Public Health and the Direction of Innovation, Equity and Participation of the Government of Aragon. 

## 3. Results

A total of 1745 students completed the questionnaire. Mean age was 13.03 years (SD 0.82; range 12–16). 54.57% of our respondents were female. Almost 20% of our sample were not enrolled in the right course for their age. Most of the students lived with their mother and/or father. Over 33% of the mothers and over 50% of the fathers had a vocational qualification or a university degree. The vast majority of the students declared themselves to be in good or excellent health. With regard to their life skills, two thirds of students had high level social skills, almost 50% of the participants had high level cognitive skills, and almost 90% of the students demonstrated a positive affective balance. In terms of their academic performance, the students’ average grade was 6.75 out of 10 (Table 1).

We analyzed the association between cognitive, social, and affective skills and academic performance. We found a moderate correlation between cognitive and social skills in the general study population, which was stronger in boys. There was a weak correlation between affective balance and academic performance, especially in the case of boys (Table 2).

With regard to sex differences in the study variables, although most of our participants had good social skills, more girls than boys achieved a high level. Boys made more of an effort to meet new people and to persuade them that their own ideas are better and more useful than those of other people. Girls, on the other hand, introduced their friends to other people, told other people that they liked them and paid more attention to instructions and explanations. In addition, girls were more likely to apologize than boys. We observed a high level of cognitive skills in our sample, but more boys than girls had high cognitive skills. Boys thought themselves more capable of overcoming unforeseeable situations thanks to their personal qualities and considered that they had the necessary abilities to manage difficult situations more so than girls. In terms of their affective skills, more than three quarters of our sample showed a positive affective balance although there were more boys than girls in this group. Girls achieved a high score in 6 out of the 9 negative affective skills, including feeling annoyed by someone, feeling lonely or removed from other people, being afraid of the future, feeling depressed or unhappy, feeling tired, feeling nervous and overwhelmed, and feeling like crying. Boys, on the other hand, achieved higher scores in the positive affective skills including feeling full of energy and feeling secure about the future (Table 3).

There was a significant association (*p* < 0.001) between academic performance and the level of social and cognitive skills in our participants. Excellent outcomes were more frequently found among students with high levels of these skills. We also found a significant relationship between positive affective balance and academic performance. These participants achieved greater proportions of good and excellent academic results. (Table 4).

## 4. Discussion

The aim of this study was to determine the life skills of 7th and 8th grade middle school students, including social skills, cognitive skills, and affective skills, and to analyze the relationship between these skills and academic performance. As a secondary aim, we analyzed gender differences in the study variables. 

The students perceived their health status as mostly good or excellent. Our results are in agreement with those of a previous, similar study [32]. This perception of their own health may be influenced by a classic understanding of the concept of health, as the absence of disease, as adolescents are frequently free from illness.

According to Caballo [4], those adolescents who have better social skills should be able to more successfully face day-to-day challenges and difficulties than those whose social skills are worse. Our findings show that nearly two thirds of our participants had good social skills. Our results differ from those obtained in preceding investigations [3,21,22], whose participants whose achieved lower social skill levels. Having said this, gender differences in the adolescents’ social skills were also found in previous investigations [20,36,37]. Specifically, girls tended to have higher social skills level; boys had more initiative to meet new people and showed a higher capacity for persuasion, whereas girls were more complacent, attentive, and assumed their mistakes [20,36]. These results may be influenced by gender stereotypes assigning boys a more competitive role and girls more empathy and assertiveness [20,36]. 

According to Bandura [38], a high level of self-efficacy can increase motivation and academic achievement. As opposed to previous investigations [3,25], we observed a high level of perceived self-efficacy in our sample. In addition, our results suggest that boys have higher levels of self-efficacy than girls. This is in agreement with previous studies [37,38,39,40], which observed that boys had more self-confidence and self-efficacy than girls. Other authors [41,42], however, did not find significant gender differences. 

Our participants’ affective balance was mostly positive, however, there were gender differences that are worth highlighting in our sample. Specifically, more boys than girls had a positive affective balance. This is in agreement with previous studies [39,43,44]. We also observed that girls experienced some of the negative variables more frequently than boys, namely loneliness, fear, unhappiness, tiredness, nervousness, and feeling like crying. Boys, on the other hand, frequently felt energized and secure. Again, our findings may be influenced by learnt gender stereotypes assigning girls a lesser degree of autonomy and a low level of subjective wellness. 

We found a significant association between life skills and academic performance. Firstly, as in previous studies [19], we observed a relationship between social skills and academic performance. Other authors [18] have found a relationship between specific social skills, such as empathy and teamwork, and academic performance. However, according to Oyarzún et al. [20], the association between these variables remains unclear. Secondly, we observed a significant association between the level of self-efficacy and academic performance. Our findings are in agreement with previous studies [8,26,45,46] that suggest that the higher the level of self-efficacy, the better the academic performance. According to Schunk [47], those students who have a higher level of self-efficacy also tend to be more motivated, have higher aspirations, and to be more prepared to work hard to fulfil them [38]. Thirdly, we found a positive association between affective balance and academic performance [27,28,29,48]. This association can be explained by the idea that subjective wellness promotes the achievement of one’s personal goals, including academic ones. Thus, a positive affective balance might improve one’s mood and state of mind which, in turn, may contribute to achieving academic success [27]. Also, it is likely that achieving high academic scores also increases personal satisfaction and happiness which, subsequently, would have an impact on subjective wellness [29]. 

Based on the above, we conclude that a positive association exists between life skills and academic achievement in adolescents. Therefore, we suggest that interventions and activities aimed at improving the life skills of children and adolescents should be integrated into school curricula in order to increase their personal satisfaction and wellbeing and support academic success. In addition, it is possible that these interventions and activities also contribute to improving health outcomes in this population both in the short- and long-term. Health promoting schools integrate health determinants and life skills into their curricula [15] and, thus, it is possible that their model contributes to improving the academic performance of their students [15,49,50]. Future studies in this area should analyze this educational model and compare their academic and other outcomes with those of non-health promoting schools with similar characteristics.

### Limitations

We wish to acknowledge a number of limitations to our study. Firstly, cross-sectional studies determine exposure and outcome at the same time and, therefore, cannot establish causality. In addition, our rate of response was superior to that of previous studies, but it was still quite low (34%). This may have introduced a degree of selection bias in our sample. Finally, our sample comprised students from the autonomous region of Aragon (Spain) only. In Spain, educational competencies are transferred to the regional governments and, therefore, our results may not be entirely applicable to the rest of the Spanish population. 

## 5. Conclusions

Our results suggest that there is a positive association between life skills and academic performance. Education and training in life skills should be integrated into curricula across all levels of compulsory education. Educational policies should adopt a health promotion approach in order to promote academic performance in the short- and medium-term, and better health outcomes and lifestyle in the medium- and long-term. Future research in this area, including longitudinal studies, should address these issues in the population of school-age children and adolescents. 

## Figures and Tables

**Table 1:** Sociodemographic characteristics and overall results from the assessment of the life skills and academic performance.

Variable	Category	N	%
Sex	Boys Girls	791 950	45.43 54.57
Grade	7th 8th	899 846	51.52 48.48
Right course for their age	Yes No	1441 304	82.58 17.42
Number of siblings	0 1 2 3 >3	293 1066 278 69 37	16.81 61.16 15.95 3.96 2.12
Sibling position	Oldest Middle Youngest	663 146 631	46.01 10.13 43.79
Co-habitants	Mother Father Mother’s partner Father’s partner Grandmother Grandfather Others Brothers Sisters	1680 1446 108 30 108 62 37 766 721	96.89 83.39 6.20 1.72 6.20 3.56 2.12 46.97 44.21
Father’s level of education	No schooling completed Primary education Secondary education Vocational training Higher education	36 190 428 553 412	2.22 11.74 26.44 34.16 25.45
Mother’s level of education	No schooling completed Primary education Secondary education Vocational training Higher education	25 118 392 521 606	1.50 7.10 23.59 31.35 36.46
Perceived health	Excellent Good Average Poor	680 945 104 3	39.26 54.56 6.00 0.17
Level of social skills	High Medium-high Medium Medium-low	1067 536 66 5	67.74 32.02 3.94 0.30
Level of cognitive skills	High Medium Low	727 450 507	43.2 26.7 30.1
Affective balance	Positive Negative	1492 189	88.76 11.24
Academic performance	Insufficient (0–4.9/10) Sufficient (5–5.9/10) Good (6–6.9/10) Excellent (7–8.9/10) Outstanding (9–10/10)	157 329 421 621 213	9.02 18.90 24.18 35.67 12.23

**Table 2:** Correlation between cognitive, social and affective skills and academic performance.

Variables	Cognitive Skills	Social Skills	Affective Balance	Academic Performance
Boys				
Cognitive skills	1	0.535 **	0.347 **	0.209 **
Social skills	0.535 **	1	0.342 **	0.184 **
Affective balance	0.347 **	0.342 **	1	0.172 **
Academic performance	0.209 **	0.184 **	0.172 **	1
Girls				
Cognitive skills	1	0.498 **	0.405 **	0.235 **
Social skills	0.498 **	1	0.427 **	0.222 **
Affective balance	0.405 **	0.427 **	1	0.231 **
Academic performance	0.235 **	0.222 **	0.231 **	1
Boys and Girls				
Cognitive skills	1	0.503 **	0.381 **	0.214 **
Social skills	0.503 **	1	0.366 **	0.218 **
Affective balance	0.381 **	0.366 **	1	0.174 **
Academic performance	0.214 **	0.218 **	0.174**	1

Spearman’s rank correlation coefficient ** *p* < 0.05 (bilateral).

**Table 3:** Statistically significant gender differences in social, cognitive, and affective skills.

Variables	Boys N (%)	Girls N (%)	Total N (%)	*p* Value
Social Skills				
Global scale Medium-low level Medium-high level Medium level High level	1 (0.1) 271 (34.3) 33 (4.2) 451 (57)	4 (0.4) 265 (27.9) 33 (3.5) 612 (64.4)	5 (0.3) 536 (30.8) 66 (3.8) 1063 (61.1)	0.019
1. Do you pay attention to the person that is talking to you and make an effort to understand what s/he is saying?				
Never Seldom Sometimes Frequently Always	2 (0.3) 14 (1.8) 114 (14.7) 373 (48.1) 272 (35.1)	2 (0.2) 19 (2) 89 (9.4) 431 (45.7) 402 (42.6)	4 (0.23) 33 (1.9) 203 (11.8) 804 (46.8) 674 (39.23)	0.002
6. Do you take the initiative to meet new people?				
Never Seldom Sometimes Frequently Always	27 (3.5) 110 (14.1) 192 (24.6) 248 (31.8) 202 (25.9)	37 (3.9) 104 (11) 209 (22.1) 278 (22.1) 317 (33.5)	64 (3.7) 214 (12.4) 401 (23.3) 526 (30.5) 519 (30.1)	0.008
7. Do you introduce your friends to other people?				
Never Seldom Sometimes Frequently Always	56 (7.2) 144 (18.4) 218 (27.9) 200 (25.6) 163 (20.9)	35 (3.7) 112 (11.9) 261 (27.6) 290 (30.7) 247 (26.1)	91 (5.3) 256 (14.8) 479 (27.8) 490 (28.4) 410 (23.8)	0.000
8. Do you tell people what you like about them or about what they do?				
Never Seldom Sometimes Frequently Always	80 (10.3) 153 (19.7) 228 (29.3) 205 (26.4) 111 (14.3)	31 (3.3) 136 (14.4) 274 (29) 303 (32.1) 201 (21.3)	111 (6.4) 289 (16.8) 502 (29.2) 508 (29.5) 312 (18.1)	0.000
12. Do you pay attention to instructions, ask for explanations, and follow instructions correctly?				
Never Seldom Sometimes Frequently Always	17 (2.2) 47 (6) 209 (26.8) 283 (36.3) 223 (28.6)	10 (1.1) 56 (6) 193 (20.5) 425 (45.2) 256 (27.2)	27 (1.6) 103 (5.99) 402 (23.4) 708 (41.2) 479 (27.9)	0.001
13. Do you apologize when you know that you have done something wrong?				
Never Seldom Sometimes Frequently Always	8 (1) 33 (4.2) 106 (13.6) 245 (31.5) 386 (49.6)	17 (1.8) 26 (2.8) 99 (10.5) 252 (26.8) 547 (58.1)	25 (1.5) 59 (3.4) 205 (11.9) 497 (28.9) 933 (54.3)	0.002
14. Do you try to persuade others that your own ideas are better and more useful than the ideas of others?				
Never Seldom Sometimes Frequently Always	87 (11.2) 178 (23) 237 (30.6) 169 (21.8) 104 (13.4)	135 (14.4) 275 (29.3) 281 (30) 178 (19) 69 (7.4)	222 (12.96) 453 (26.4) 518 (30.2) 347 (20.3) 173 (10.1)	0.000
Cognitive skills/self-efficacy				
Global scale Low level Medium level High level	206 (27.1) 200 (26.4) 353 (46.5)	301 (32.5) 250 (27) 374 (40.4)	507 (30.1) 450 (26.7) 727 (43.2)	0.022
5. Thanks to my personal qualities and resources I am able to overcome unforeseeable situations.				
Untrue of me Somewhat untrue of me Somewhat true of me True of me	25 (3.2) 89 (11.4) 396 (50.8) 270 (34.6)	19 (2) 168 (17.9) 448 (47.6) 306 (32.5)	44 (2.6) 257 (14.9) 844 (49) 576 (33.5)	0.001
6. When I find myself in a difficult situation I can remain calm because I have the necessary abilities to manage difficult situations.				
Untrue of me Somewhat untrue of me Somewhat true of me True of me	44 (5.6) 226 (42) 335 (42.9) 175 (22.4)	117 (12.4) 312 (32.9) 375 (39.6) 143 (15.1)	161 (9.3) 538 (31.2) 710 (41.1) 318 (18.4)	0.000
7. I am generally capable of managing any situation.				
Untrue of me Somewhat untrue of me Somewhat true of me True of me	29 (3.7) 179 (23) 421 (54.1) 149 (19.2)	27 (2.9) 273 (28.9) 479 (50.6) 167 (17.7)	56 (3.2) 452 (26.2) 900 (52.2) 316 (18.3)	0.044
8. I can solve most problems if I make an effort.				
Untrue of me Somewhat untrue of me Somewhat true of me True of me	15 (1.9) 58 (7.4) 252 (32.2) 457 (58.4)	7 (0.7) 72 (7.6) 366 (38.6) 502 (53)	22 (1.3) 130 (7.5) 618 (35.7) 959 (55.5)	0.008
9. If I am in a difficult situation, I generally know what to do.				
Untrue of me Somewhat untrue of me Somewhat true of me True of me	30 (3.9) 134 (17.2) 393 (50.5) 221 (28.4)	31 (3.3) 222 (23.5) 479 (50.7) 213 (22.5)	61 (3.5) 356 (20.7) 872 (50.6) 434 (25.2)	0.002
Affective skills				
Global Scale Positive Negative	701 (88.6) 58 (7.3)	787 (82.8) 131 (13.8)	1488 (85.5) 189 (10.9)	0.000
1. Have you felt annoyed by someone?				
Never or almost never Sometimes Always or almost always	234 (30) 465 (59.6) 81 (10.4)	224 (23.7) 595 (62.9) 127 (13.4)	458 (26.5) 1060 (61.4) 208 (12.1)	0.005
2. Have you felt very lonely or removed from other people?				
Never or almost never Sometimes Always or almost always	537 (68.8) 206 (26.4) 38 (4.9)	544 (57.6) 321 (34) 49 (8.4)	1081 (63.8) 527 (31.1) 87 (5.1)	0.000
3. Have you felt that things were going your way?				
Never or almost never Sometimes Always or almost always	70 (9) 421 (54.1) 287 (36.9)	103 (11) 580 (61.7) 257 (27.3)	173 (10.1) 1001 (58.3) 544 (31.7)	0.000
4. Have you felt very worried?				
Never or almost never Sometimes Always or almost always	199 (25.6) 440 (56.7) 137 (17.7)	191 (20.2) 507 (53.7) 247 (26.1)	390 (22.7) 947 (55) 384 (22.3)	0.000
6. Have you been afraid of what may happen?				
Never or almost never Sometimes Always or almost always	204 (26.3) 445 (57.4) 126 (16.3)	152 (16.1) 558 (59.2) 232 (24.6)	356 (20.7) 1003 (58.4) 358 (20.9)	0.000
8. Have you felt depressed or very unhappy?				
Never or almost never Sometimes Always or almost always	458 (59.2) 249 (32.2) 67 (8.7)	479 (51) 340 (36.2) 121 (12.9)	937 (54.7) 589 (34.4) 188 (11)	0.001
9. Have you felt full of energy?				
Never or almost never Sometimes Always or almost always	23 (3) 231 (30) 515 (67)	42 (4.5) 348 (37) 551 (58.6)	65 (3.8) 579 (33.9) 1066 (62.3)	0.001
10. Have you felt very tired?				
Never or almost never Sometimes Always or almost always	151 (19.5) 379 (49) 243 (31.4)	147 (15.7) 522 (55.6) 270 (28.8)	298 (17.4) 901 (52.6) 513 (30)	0.017
11. Have you felt very nervous, overwhelmed or tense?				
Never or almost never Sometimes Always or almost always	185 (24) 409 (53) 177 (23)	141 (15) 513 (54.6) 285 (30.4)	326 (19.1) 922 (53.9) 462 (27)	0.000
14. Have you felt like crying?				
Never or almost never Sometimes Always or almost always	298 (38.7) 352 (45.7) 121 (15.7)	253 (26.9) 479 (51) 208 (22.1)	551 (32.2) 831 (48.6) 329 (19.2)	0.000
16. Have you felt secure with regard to the future?				
Never or almost never Sometimes Always or almost always	121 (15.7) 387 (50.3) 262 (34)	210 (22.4) 466 (49.8) 260 (27.8)	331 (19.4) 853 (50) 522 (30.6)	0.000

**Table 4:** Association between life skills and academic performance.

Variables	Insufficient N (%)	Sufficient N (%)	Good N (%)	Excellent N (%)	Outstanding N (%)	*p* Valor
Social skillsMedium-low level Medium-high level Medium level High level	3 (1.9) 62 (39.5) 6 (3.8) 81 (51.6)	0 (0) 25 (7.6) 114 (34.7) 167 (50.8)	1 (0.2) 20 (4.8) 141 (33.5) 246 (58.4)	0 10 (1.6) 148 (23.7) 450(72.1)	1 (0.5) 5 (2.3) 71 (33.2) 123 (57.5)	0.000
Cognitive skills Low level Medium level High level	31 (46.7) 36 (23.7) 45 (26.9)	115 (36.5) 75 (23.8) 125 (39.7)	129 (31.7) 120 (29.5) 158 (38.8)	131 (21.7) 164 (27.1) 310 (51.2)	61 (29.2) 57 (27.3) 91 (43.5)	0.000
Affective skills Positive affective balance Negative affective balance	123 (78.3) 33 (21)	263 (79.9) 42 (12.8)	352 (83.6) 54 (12.8)	558 (89.4) 50 (8)	196 (91.6) 10 (3.7)	0.000

## Data Availability

The anonymous data presented in this study are available upon request from the first author. The data are not publicly available due to the legislation on personal data protection and other current legislation.
